# Supplementary material for: Factors influencing community acceptability of mass drug administration for the elimination of onchocerciasis in the Asante Akim South Municipal, Ghana
Source: PLoS Negl Trop Dis. 2023 Mar 31;17(3):e0011251. doi: 10.1371/journal.pntd.0011251 (PMC10096254; doi:10.1371/journal.pntd.0011251)
Supplement: Attachment — Submitted filename: RESPONSE TO REVIEWER COMMENTS.docx [file pntd.0011251.s001.docx]

Dear Editor,

**RESPONSE TO REVIEWERS’ COMMENTS**

We would like to thank you and the reviewers for the thorough review of our manuscript and the invaluable comments/recommendation offered for improving the overall quality of the paper.

We have addressed all the issues raised and believe the current version of the paper meets the journal’s publication requirements.

We are very grateful for this wonderful opportunity to publish in your highly esteemed journal.

Please find below our responses to the issues raised:

**Methods**

**Reviewer #1**

**Comment:** The authors clearly stated the objective of the study. Authors have obtained permission from the appropriate ethic regulatory bodies for the study. However, there is no mention of whether permissions were sought from the chiefs and opinion leaders from each of the communities where the study was conducted. I suggest this information is included in the manuscript

**Response**: Thank you very much for such an important observation. Please we sought permission from the community leaders including the chiefs and have included this information under the Ethics statement section. Please see lines 159-162 of the revised manuscript for this included information.

**Comment**: Please let the ‘ethical consideration’ statement appear at the first section under the methods and not to the end

**Response**: Please this has been done accordingly and could be found from line 155 to 162 of the revised paper.

**Comment**: The authors have not indicated what informed them to choose/select the study site. No mention of the endemicity of the infection, ivermectin mass drug administration activities if there is any, the coverage, compliance to ivermectin in previous or a recent mass drug administration (MDA). Please I suggest the authors include these details in the study design and setting to be able to address the stated objective.

**Response**: Please we chose the study area because it is one of the endemic areas for onchocerciasis in Ghana. Also, previous reports have indicated coverage rate of MDA below 80%, as well as some of the community members refusing to accept and ingest ivermectin administered/distributed to them. This information has been included under the Study Design and Setting section from line 165 to 169 of the revised manuscript.

**Comment**: The authors clearly described the appropriate population suitable for the study. However, the exclusion criterium in line 139 is not clear and should be stated with clarity

**Response:** Please we have restated the exclusion criteria for clarity. It now reads “Community members below the age of 18 years and persons having mental problems were excluded from the study” (Lines 181 and 182 of the revised manuscript).

**Comment**: There is no mention of seeking for consent from the participants before they were enrolled into the study. I suggest that this information, either written or orally obtained should be included in the study instrument and data collection section.

**Response**: Please we sought consent orally from the participants before administering the questionnaire and this information has been included in the revised manuscript as suggested. Please see lines 246 and 247.

**Comment:** The authors have not mentioned the statistical test used to draw conclusion from the data. I suggest this important information is included under the data processing and analysis.

**Response**: Please the statistical test used is a Multiple linear regression analysis and this has been stated and highlighted at the data processing and analysis section (line 261), as well as under Table 5.

**Reviewer #2:**

**Comment**: This is a very well written and presented manuscript on an important topic. The objectives and methods are clearly presented; there was not a clear hypothesis, but I do not view that as a deficiency. Description of study population, sample size and statistical analyses are good. I have no concerns about ethical approvals.

**Response**: We are very grateful for this wonderful observation.

**Results**

**Reviewer #1**

**Comment**: The results are not completely presented. I suggest that Table 5 is edited to include the
following:
- a column containing information on the P- values
- the third column that indicate the “B/ β”, should be written in full and write
“range” in bracket to let the readers understand the table without referring to the
manuscript
- in the results section of the manuscript, indicate the p values computed instead of
just writing ‘p’ is greater/lower than 0.05
- add to the footnote the statistical test used to calculate the p values

**Response**: Please we have edited Table 5 to include a column containing the p values, and the third column indicating B now written “Standardized regression coefficient (range)”. In the manuscript, the p values computed have been indicated. We have also added to the footnote of Table 5 the multivariable or multiple linear regression test used to generate the p values.

**Comment**: Table 2 & 5 are overlapping. I suggest that if a table cannot fit into one page, the authors should break that into sections. Example; Table 1, Table 1 (continued) maintaining the
title of the columns under each table

**Response**: Please both Table 2 and 5 have been split into two to avoid the overlap.

**Reviewer #2:**

**Comment**: Well described and tables are simple and clear.

**Response**: We are grateful for the observation and comment.

**Conclusions**

**Reviewer #1:**

The authors provided conclusions which are supported by the data presented in the manuscript.
The limitations of the study were clearly described.
The benefit of the findings to the advancement of knowledge on the topic under study have been
discussed and public health relevance has also been touched on.

**Response**: We are very grateful for the observation and comments

**Reviewer #2**

Conclusions and limitations well presented, and public health relevance is obvious (increased education and information distribution). I believe that the results will be beneficial to future MDA programs for oncocerciasis and other diseases

**Response**: We are very grateful for the observation and comments

**Editorial and Data Presentation Modifications**

**Reviewer #1**

**Comment**: As commented under the result section, I suggest that tables in the manuscript that cannot fit into a page, should be broken down into sections to avoid overlapping.

**Response**: Please we have done the revision as indicated in the response to comment #1 under Results above

**Comment**: Table 5 should be edited to include the fields mentioned under the result section

**Response**: Please this has been done accordingly

**Reviewer #2**

**Comment**: I don't believe the word Ivermectin needs to be or should be capitalized

**Response**: Please this has been corrected accordingly throughout the revised manuscript

**Comment**: It appears that, likely through editing, multiple font sizes were used - please make font size consistent

**Response**: Please we have revised the manuscript to make the font size 12 throughout

**Comment**: Line 355: aassociated -> associated
**Response**: Please this has been corrected accordingly

**Summary and General Comments**

**Reviewer #1**

**Comment:** The manuscript has not been edited and it contains different font sizes. I suggest authors edit the manuscript to have a uniform font size

**Response**: Please the font sizes have been edited accordingly. Font size 12 has now been used throughout the manuscript.

**Comment**: The in-text citation is incorrect. The journal requires a square bracket not parenthesis

**Response**: Please we have revised the in-text citation accordingly.

**Comment:** The authors should please provide citation to the statement made in line 83-86.

**Response:** Please this has been done accordingly. Please see line 117-120 of the revised manuscript for the citation provided.

**Comment**: I suggest the authors be specific on the kind of mass media being referred to at the end of line 88 and 89

**Response:** Please the kind of mass media being refer to are radio, television and newspapers. This information has been included in the revised manuscript. Please see lines 119 and 120 for this information.

**Comment**: • In line 92 and 93, the authors suggested low acceptability of MDA based on the
prevalence of a study stated in line 90 to 92. To make their suggestion more convincing, I
will recommend they mention the IVM coverage or compliance recorded in that study

**Response:** Please the aim of the study (Reference No. 12) was to determine the prevalence of onchocerciasis and associated clinical conditions frequently associated with the disease in endemic communities in Ghana. We suggested that the high prevalence reported by the study might be due to low acceptability of MDA in the endemic communities and supported our suggestion with two studies: Reference No. 13 which found that 33.3% of the eligible population did not comply with the MDA program; and Reference No. 14 which observed that 63.9% of the respondents did not receive the drug (ivermectin) during the 2019 MDA program and more than half were not aware of the program. Thus, the study (Ref. No. 12) did not report on IVM coverage. It rather reported on the prevalence of onchocerciasis, to indicate the existence, of the disease in some Ghanaian communities despite the implementation of MDA programs over the years.

**Comment:** Remove the letter “s” from the word “treatment’s” in line 111
**Response:** Please this has been corrected accordingly

**Comment:** Line 210 and 211. How were the data checks done, who did it? I recommend the authors elaborate on this please

**Response:** Completeness of the data was checked by two members of the research team who supervised four research assistants we trained and used as data collectors. The two members of the research team checked every section of each filled questionnaire to ensure completeness. A third member of the research team also checked the filled questionnaires before coding was done. Please see line 251-255 for the addition of this information to the revised manuscript.

**Comment:** In line 215, the authors mentioned that the reliability of the items measuring the
composite variable was tested without mentioning how it was done. I suggest this should
be elaborated on

**Response:** Please we used Cronbach’s alpha to test the reliability of the items measuring the
composite variable (i.e., acceptability of ivermectin MDA). We did this to see if there was any correlation between the nine items before combining them into a single indicator. The alpha value was high (0.89), thus indicating a high internal consistency reliability. Line 258 -260 of the revised manuscript provide this information. We used STATA software to generate the Cronbach’s alpha value of the nine items.

**Comment:** There is no numbering of the write-up after Table 2

**Response:** Please this has been revised accordingly

**Comment:** What do the authors mean by using the word “significant” in the beginning of the second sentence after Table 2? Was the statement based on statistically significant level. I
suggest the sentence should be rephrased. Revised to most of them ………….

**Response:** Please we have accordingly replaced “significant” with “Most of them”

**Comment:** Add colon sign after the “Table 4” in the Table 4 heading (i.e., Table 4:).

**Response:** Please this has been done accordingly

**Comment:** Replace “reeducation” in line 327 with “reduction”.

**Response:** Please this has been done accordingly

**Comment:** No mention of authors contribution in the manuscript. I suggest authors include this
information after “Funding”

**Response:** Please authors contribution statement has been included in the revised manuscript after the Funding statement.

**Comment:** Some of the bibliographies are incorrect. For example, “World Health Organization”
should either be written in full or abbreviated as “WHO” but not “World Health O”. I
recommend that the authors edit the bibliography to suite the journal’s style

**Response:** Please the bibliographies have been revised accordingly to suite the journal’s style.

Thank you
