# Supplementary material for: Factors influencing community acceptability of mass drug administration for the elimination of onchocerciasis in the Asante Akim South Municipal, Ghana
Source: PLoS Negl Trop Dis. 2023 Mar 31;17(3):e0011251. doi: 10.1371/journal.pntd.0011251 (PMC10096254; doi:10.1371/journal.pntd.0011251)
Supplement: Attachment — Submitted filename: RESPONSE TO REVIEWER COMMENTS.docx [file pntd.0011251.s002.docx]

Dear Editor,

**RESPONSE TO REVIEWERS’ COMMENTS**

We once again thank you and the reviewers for the thorough review of the first revised version of this manuscript and the invaluable comments/recommendation offered for improving the overall quality of the paper.

We have addressed all the issues raised and believe the current version of the paper meets the journal’s publication requirements.

We are very grateful for this wonderful opportunity to publish in your highly esteemed journal.

Please find below our responses to the issues raised:

**Methods**

**Reviewer #1**

**Comment:** Line 160-162: Kindly be specific on the type of inform consent sought from the community leaders (that is either verbal or   written)

**Response**: Please we sought verbal consent from the community leaders and this has been added to the revised manuscript. Please see line 160 of the revised paper for this information.

**Comment**: Line 165-168: Please for how long has MDA activities/programs be in place in the selected municipality? Kindly mention this.

**Response**: Please MDA activities have been in place in the municipality since 1995. This has been added to the revised version of the paper. Please see lines 169 and 170 for this additional information added.

**Comment**: Line 249: Kindly end the sentence with the word “period” to read “collection period”

**Response:** Please this has been done accordingly.

**Results**

**Reviewer #1**

**Comment**: Table 2:  Kindly add “s” to the word “Characteristic” to read “Characteristics”
For the second Table 2 heading, kindly insert the "(continued)" after Table 2 to read “Table 2 (continued): Characteristics of the study respondents” but not at the end of the title. Please do same for the second Table 5 as well

**Response**: Please all these have been corrected accordingly.

Thank you very much. We are very grateful to you and the reviewers for helping us improve the overall quality of the paper. Their comments have been very encouraging and helpful.
